# Spectrally Shaped DP-16QAM Super-Channel Transmission with Multi-Channel Digital Back-Propagation

**DOI:** 10.1038/srep08214

**Published:** 2015-02-03

**Authors:** Robert Maher, Tianhua Xu, Lidia Galdino, Masaki Sato, Alex Alvarado, Kai Shi, Seb J. Savory, Benn C. Thomsen, Robert I. Killey, Polina Bayvel

**Affiliations:** 1 Optical Networks Group, University College London, Torrington Place, London WC1E 7JE, United Kingdom; 2 NEC Corporation, Abiko, Japan

## Abstract

The achievable transmission capacity of conventional optical fibre communication systems is limited by nonlinear distortions due to the Kerr effect and the difficulty in modulating the optical field to effectively use the available fibre bandwidth. In order to achieve a high information spectral density (ISD), while simultaneously maintaining transmission reach, multi-channel fibre nonlinearity compensation and spectrally efficient data encoding must be utilised. In this work, we use a single coherent super-receiver to simultaneously receive a DP-16QAM super-channel, consisting of seven spectrally shaped 10GBd sub-carriers spaced at the Nyquist frequency. Effective nonlinearity mitigation is achieved using multi-channel digital back-propagation (MC-DBP) and this technique is combined with an optimised forward error correction implementation to demonstrate a record gain in transmission reach of 85%; increasing the maximum transmission distance from 3190 km to 5890 km, with an ISD of 6.60 b/s/Hz. In addition, this report outlines for the first time, the sensitivity of MC-DBP gain to linear transmission line impairments and defines a trade-off between performance and complexity.

The demand for global IP communication bandwidth is continuously growing, currently estimated at 21% per year, with the growth of mobile and video data as the key drivers^1^. Optical fibre transports over 95% of all digital data traffic and was traditionally viewed as a medium with unlimited capacity. However, due to the impact of fibre nonlinearities, the capacity increases in wavelength division multiplexing (WDM) research have slowed to approximately 20% per year over the last decade^2^,^3^. The fundamental limit to the nonlinear channel is unknown to this date and is a subject of investigation. Therefore, recent research on maximising the single fibre core capacity has focused on increasing the information spectral density of each WDM channel, while simultaneously employing advanced coding and fibre nonlinearity mitigation techniques to maximise the achievable transmission distance.

The ISD of an optical network can be increased by using advanced modulation formats with high cardinality and by reducing the frequency spacing between WDM channels; however both techniques are accompanied by significant limitations. For example, increasing the cardinality of the modulation format comes at the expense of requiring a higher signal to noise ratio (SNR), which places stringent demands on the transmitter and receiver subsystems. Alternatively, as the frequency spacing between WDM channels is reduced, inter-channel interference begins to cause significant performance penalties due to linear crosstalk. Although tight filtering can be employed to constrain the bandwidth (BW) of each WDM channel, the filtering process itself results in significant inter-symbol interference (ISI) within each channel. However, if an appropriate filter shape is used, for example a *sinc* shaped pulse with a corresponding rectangular spectrum, then the Nyquist criterion for ISI can be met. A raised cosine (RC) filter satisfies the Nyquist criterion and is a common filter employed to limit the bandwidth of WDM channels. A root-raised cosine (RRC) filter is typically employed at the transmitter, with a corresponding matched RRC filter at the receiver. Only after matched filtering is the overall RC spectral shape realised, thus ensuring that the maximum of each impulse coincides with the zeros of the adjacent pulses, thereby avoiding ISI. Additionally, by decreasing the roll-off factor of the RRC filters, the WDM channel spacing can be reduced closer to the Nyquist frequency, without incurring significant penalties due to linear crosstalk^4^.

Spectral filtering has been previously performed in the electrical, optical and digital domains, and it has been demonstrated that digital filtering currently provides the optimum performance^5^. Nyquist-spaced WDM transmission was achieved using the DP-QPSK modulation format with an ISD of 3.6 b/s/Hz^6^, while ISD's of 6.25 b/s/Hz and 8.67 b/s/Hz have been achieved for DP-16QAM^7^ and DP-64QAM^8^, respectively. For DP-16QAM transmission systems, the channel spacing has been reduced to values as low as 102.5% of the symbol rate^7^ and various Nyquist filter roll-off factors have been reported, ranging from 30% down to 0.1%^9^. Single channel digital back-propagation (SC-DBP)^10^,^11^ and a concatenated soft decision forward error correction (SD-FEC) decoder have also been incorporated in ref. 9 to increase the reach of a DP-16QAM transmission system to 9100 km, while simultaneously achieving a high ISD of 6 b/s/Hz. DBP has typically been demonstrated experimentally only on a single WDM channel (SC-DBP) due to the electrical bandwidth limitations of the digital coherent receiver. However, Fontaine et al.^12^ demonstrated the use of a spectrally sliced coherent receiver to achieve an optimal Q^2^-factor gain of 1 dB by back-propagating a 5-channel 30 GBd DP-16QAM super-channel, which was transmitted over a fixed distance of 960 km. A single coherent super-receiver was used to back-propagate a 4-channel 28 GBd orthogonal frequency division multiplexed super-channel and an enhanced Q^2^-factor margin was achieved at a fixed distance of 1000 km^13^. The ability to receive and digitally back-propagate multiple WDM channels in a single-receiver, known as multi-channel digital back-propagation (MC-DBP)^14^, can give a significant advantage in providing efficient and effective nonlinearity mitigation as MC-DBP can compensate for cross-channel nonlinear impairments, rather than just self-phase modulation as in SC-DBP.

In this paper, we experimentally investigate the optimum roll-off factors of the transmitter and receiver RRC filters as a function of the WDM channel spacing, in order to mitigate linear crosstalk induced penalties in a Nyquist spaced 7-channel 10 GBd DP-16QAM transmission system. A single digital coherent super-receiver is used to simultaneously receive and demodulate the entire super-channel and MC-DBP is employed to increase the maximum reach of the DP-16QAM WDM transmission system. The sensitivity of MC-DBP to transmission line impairments and the associated complexity of the technique are also discussed. The experimental results are verified analytically using the Gaussian noise (GN) model^15^,^16^ and through numerical simulations based on the nonlinear Schrödinger equation (NLSE) for this system.

## Results

### Spectrally Shaped DP-16QAM Transmission System

**Figure 1:**
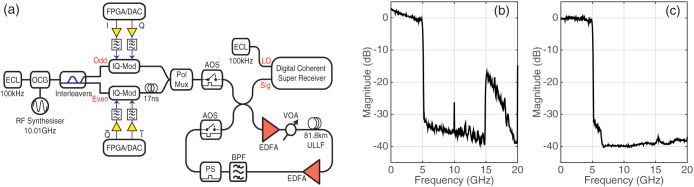
Seven channel spectrally shaped DP-16QAM transmission system. (a) Experimental setup. (b) RF spectrum of spectrally shaped 4-level signal from one of the DAC outputs, without cascaded analogue electrical low pass filtering and (c) corresponding output of the DAC with signal pre-emphasis and electrical low pass filtering.

A schematic of the 7-channel 10 GBd DP-16QAM transmission system is illustrated in Fig. 1(a). The output of an external cavity laser (ECL) with a linewidth of 100 kHz was passed through an optical comb generator (OCG) that consisted of two cascaded Mach-Zehnder modulators, both overdriven with an amplified sinusoidal wave. This generated seven, evenly spaced, frequency locked comb lines, with the channel spacing equal to the frequency of the applied sine wave. The number of comb lines was limited to seven in order to maintain a power variation of <1 dB across the comb and to ensure sufficient dynamic range in per channel launch power to demonstrate the effectiveness of nonlinearity mitigation. The frequency comb was separated into odd and even carriers using three cascaded Kylia micro-interferometer (MINT) interleavers. Each set of comb lines were independently modulated using two IQ modulators. Four decorrelated pseudo-random binary sequences (PRBS) of length 2^15^−1 were digitally generated offline and combined to provide two 4-level driving signals, which were spectrally shaped using a truncated RRC filter. The filter design was minimum order with a specified stop-band attenuation and roll-off factor. The roll-off factor ranged from 10% down to 0.1% and the number of filter taps increased from 320 for the 10% filter to ~1000 for the lowest roll-off factor of 0.1%. There was also a corresponding decrease in stop band attenuation from 40 dB down to 20 dB as the filter roll-off factor was reduced.

The RRC filtered in-phase (I) and quadrature (Q) signals were loaded onto a pair of field-programmable gate arrays (FPGAs) and output using two digital-to-analogue convertors (DACs) operating at 20 GS/s (2 samples per symbol). The RF spectrum of the in-phase electrical output from one of the DACs is shown in Fig. 1(b). There is a large roll-off evident at pass-band frequencies (0–5 GHz) caused by the electrical bandwidth limitation of the transmitter, while there is significant power in the stop-band (>5 GHz) stemming from the DAC image and an additional tone at the DAC half-clock rate (10 GHz). The signals were pre-emphasised offline to overcome the electrical response of the transmitter and the output of each DAC was passed through two cascaded electrical low pass filters (LPFs) to remove the image signal. Both LFPs were 8th order Bessel filters with rejection ratios in excess of 20 dB/GHz and had 3 dB bandwidths of 7.46 GHz and 6.2 GHz. The RF spectrum of the electrically amplified RRC filtered four-level signal applied to the IQ modulators is illustrated in Fig. 1(c). The modulated odd and even channels were decorrelated by 170 symbols before being combined and polarisation multiplexed to form a 7-channel 10 GBd DP-16QAM signal.

For back-to-back error analysis the output of the polarisation multiplexing stage was passed straight into the signal port of the coherent super-receiver. For transmission experiments, a single-span recirculating fibre loop was used. The loop consisted of two acousto-optic switches (AOS), two erbium-doped fibre amplifiers (EDFAs), each with a noise figure of 4.5 dB, an optical band-pass filter for ASE removal, loop-synchronous polarisation scrambler (NRT 2510) and a variable optical attenuator (VOA) to control the signal launch power. The fibre span consisted of 81.8 km of Corning® SMF-28® ultra low loss (ULL) fibre and the total span loss, including splices and optical connectors, was 13.4 dB. The combined loss for the remaining passive components within the recirculating loop was 13 dB.

A polarisation diverse coherent receiver with a 3 dB electrical bandwidth of 70 GHz utilised a second 100 kHz ECL as a local oscillator and the received signals were captured using a 160GS/s real-time sampling oscilloscope with 63 GHz analogue electrical bandwidth. Digital signal processing (DSP) and SD-FEC was performed offline using Matlab and is detailed in the Methods section. Numerical and analytical simulations were carried out to verify the experimental results and are also outlined in the Methods section.

### Linear Crosstalk Induced OSNR Penalty

**Figure 2:**
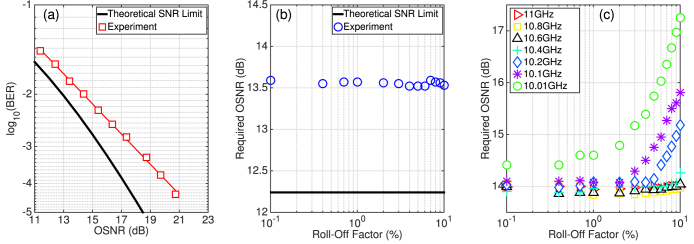
Back-to-back performance of the spectrally shaped DP-16QAM transmitter. (a) BER as a function of received OSNR (0.1 nm resolution bandwidth) for a single channel with a roll-off factor of 0.1%. (b) Required OSNR to achieve a BER of 1.5·10^−2^ as a function of the RRC filter roll-off factor for a single channel system. (c) Required OSNR to achieve a BER of 1.5·10^−2^ as a function of both the RRC filter roll-off factor and the channel spacing, for the central channel, in the 7-channel WDM system.

The back-to-back performance of the digital transmitter was initially verified using a single channel and is illustrated in Fig. 2(a). The roll-off factor of the RRC filter was set to 0.1% and an implementation penalty of 1.3 dB relative to the theoretical SNR limit was achieved at a BER of 1.5·10^−2^. The required optical signal to noise ratio (OSNR) to achieve a BER of 1.5·10^−2^, as a function of the filter roll-off factor, was experimentally measured and is shown in Fig. 2(b). The average OSNR was 13.5 dB as the roll-off factor increased from 0.1% to 10% and varied by ±0.05 dB, which was within the measurement accuracy of our system. This demonstrates that there was no intrinsic penalty associated with the RRC filter characteristics and provides a base-line level of performance for the 10 GBd DP-16QAM digital transmitter.

**Figure 3:**
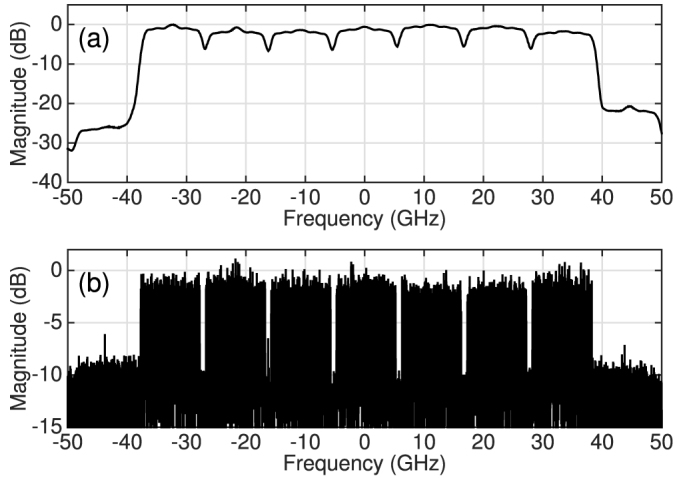
Seven channel WDM spectra. (a) Optical spectrum of 11 GHz spaced 7-channel DP-16QAM signal, measured using an OSA with a resolution of 0.01 nm. The roll-off factor of the RRC filter was set at 0.1%. (b) Corresponding PSD of the coherently detected signal.

Fig. 2(c) shows the back-to-back performance for the 7-channel DP-16QAM transmitter as a function of both the WDM channel spacing and RRC filter roll-off factor. An additional implementation penalty of 0.4 dB was measured at a channel spacing of 11 GHz and for a roll-off factor of 0.1%, which was due to the finite stop-band attenuation of the RRC digital filter in the transmitter. At this WDM channel spacing (11 GHz), the required OSNR (13.9 dB) to achieve a BER below 1.5·10^−2^ was constant as a function of the RRC filter roll-off factor. This can be intuitively understood by analysing the received spectrum of the 7-channel 16QAM signal illustrated in Fig. 3. The optical spectrum of the 7-channel signal is shown in Fig. 3(a) and was recorded using an optical spectrum analyser (OSA) with a 0.01 nm resolution. The seven WDM channels exhibited a power variation of <2 dB and the unwanted out of band comb lines were suppressed by ~20 dB. The limited resolution of the OSA was overcome by computing the power spectral density (PSD) of the coherently received signal, as shown in Fig. 3(b). As the highest considered roll-off factor was 10%, the optical bandwidth did not exceed 11 GHz, therefore no linear crosstalk induced OSNR penalty was experienced at this spacing.

However, as the channel spacing was reduced, there was a corresponding increase in the required OSNR to achieve a BER below 1.5·10^−2^ for a RRC filter roll-off factor of 10%. This penalty was due to linear crosstalk between neighbouring WDM channels and caused the required OSNR to gradually increase to 14.3 dB as the channel spacing approached 10.4 GHz. Below this spacing there was a sharp degradation in performance, thus requiring a reduction in the channel bandwidth or decrease in the RRC filter roll-off factor. The OSNR penalty was found to reduce linearly as the RRC filter roll-off factor was decreased and an acceptable level of performance was achieved for a roll-off factor of 1% at any channel spacing greater than 10.1 GHz. However, in order to incur the minimum performance penalty due to linear crosstalk in a Nyquist spaced (10 GHz) 10 GBd DP-16QAM WDM transmitter, while simultaneously achieving the highest ISD, the roll-off factor must be reduced to 0.1%.

It is important to note, that in this work a channel spacing of 10.01 GHz was chosen for the Nyquist spaced case, as an artificial performance enhancement was experienced when the channel spacing was identical to the symbol rate. This is a common problem when employing odd and even modulated channels to represent a Nyquist spaced WDM system and has previously been observed in the literature^17^,^18^. A small shift in channel spacing (10 MHz) is sufficient to negate this unrealistic performance improvement, which was confirmed using simulations that incorporated fully decorrelated data.

### Transmission Performance with EDC

**Figure 4:**
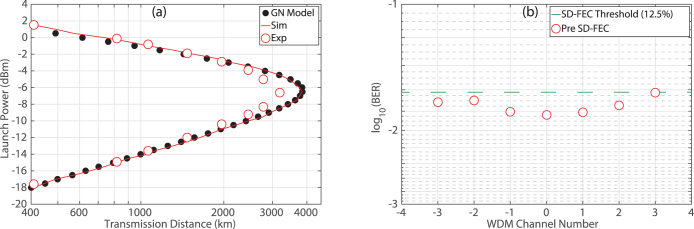
Transmission performance of the spectrally shaped DP-16QAM transmitter with EDC only. (a) The experimentally measured reach curve (open symbols) shows the maximum reach recorded to achieve a BER below 1.5·10^−2^ for a given launch power. Measurements of distance versus launch power were made on the central WDM channel. Numerical simulations (solid line) and an analytical approximation based on the GN model (closed symbols) are also displayed. (b) Received BER for all seven channels after transmission over 3190 km of SMF-28 ULL fibre.

From the back-to-back system characterisation, a channel spacing of 10.01 GHz and a RRC digital filter with a roll-off factor of 0.1% was chosen for WDM transmission as this provided the highest ISD with an acceptable implementation penalty of <2.2 dB. Fig. 4(a) illustrates the transmission performance as a function of launch power for the Nyquist spaced DP-16QAM signal with electronic dispersion compensation (EDC) only. This measurement was obtained by recording the maximum reach at a BER below 1.5·10^−2^ for a given launch power and was performed on the central WDM channel only. The analytical approximation based on the GN model (closed symbols) predicted a maximum reach of 3850 km at an optimal launch power of −6.5 dBm per channel. Excellent agreement was observed for the numerical simulations (solid line), which also indicated a maximum transmission distance of 3850 km. The experimentally measured reach curve (open symbols) demonstrated good agreement with the numerical and analytical results, in both the linear and nonlinear regions. A maximum transmission distance of 3190 km was achieved at an optimal launch power of −6.6 dBm per channel, which again is in good agreement with the GN model. The discrepancy in terms of maximum reach between the experimental points and the numerical simulation is attributed to polarisation dependent loss, which occurs in a practical system but was not included in the simulations.

Fig. 4(b) illustrates the performance of each WDM channel at the maximum transmission distance of 3190 km, with channel ‘0’ representing the central channel. All seven channels were simultaneously received using the coherent super-receiver and each individual channel was digitally down converted to baseband before the DSP and SD-FEC was performed. This ensured that the coherent receiver was operated as a true super-receiver, thus demonstrating the capability of the reception and demodulation of optical super-channels. The lowest BER of 1.49·10^−2^ was achieved for the central WDM channel (0) but increased to 1.95·10^−2^ and 2.37·10^−2^ for edge channels −3 and +3 respectively. The gradual increase in BER towards the edge of the super-channel was caused by the frequency dependent effective number of bits (ENOB) of the analog to digital convertors (ADCs) in the sampling oscilloscope. In addition to the frequency dependent ENOB, the OSNR of each channel was not identical due to the variation in power across the frequency comb generated at the transmitter, which also resulted in a slight variation in the received BER between channels.

The highest pre-FEC BER (2.37·10^−2^) was recorded for channel +3, which was corrected to below 4.7·10^−3^ using a SD-FEC code with a 12.5% overhead (OH). The post SD-FEC BER can be brought down to below approximately 10^−15^ by using a concatenated coding scheme that utilises an outer HD-FEC code with an OH of 6.25%^19^, as explained in the Methods section. Therefore, the total FEC overhead is a combination of the two concatenated codes and was 19.53%, which resulted in an ISD of 6.69 b/s/Hz and a spectral efficiency distance product (SEDP) of 21341(b·km)/s/Hz. A greater transmission reach could be achieved for a higher received BER, however this would require a larger OH FEC, thereby ultimately reducing the ISD. Therefore, in order to increase the reach of the DP-16QAM super-channel transmission system, without sacrificing ISD, we turn our attention to nonlinearity compensation.

### MC-DBP for Nonlinearity Compensation

**Figure 5:**
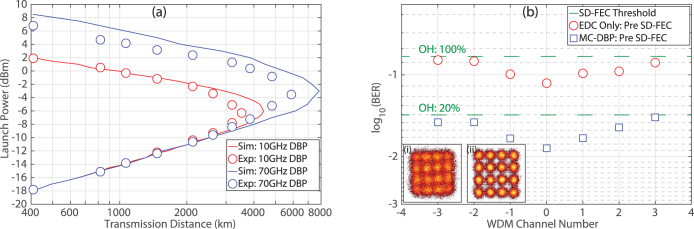
Transmission performance of the spectrally shaped DP-16QAM transmitter with MC-DBP. (a) The experimentally measured reach curve (symbols) shows the maximum reach recorded to achieve a BER below 1.5·10^−2^ for SC-DBP and MC-DBP. Numerical simulations (solid lines) are also displayed. (b) Received BER (pre SD-FEC) for all seven channels after transmission over 5890 km of SMF-28 ULL fibre for the EDC only and MC-DBP cases. The corresponding received constellations for the central channel are shown as insets (i) and (ii), respectively.

The corresponding reach curve (performed on the central channel) for the 7-channel DP-16QAM WDM signal with both single channel (10 GHz bandwidth) and multi-channel (70 GHz bandwidth) DBP is illustrated in Fig. 5(a). For single channel DBP, a maximum transmission reach of 3517 km was achieved at an optimum launch power of −6.3 dBm, thus providing a modest reach enhancement of 10% compared to when only EDC is employed in the digital coherent super-receiver. However, when the full 70 GHz bandwidth of the optical super-channel was simultaneously back-propagated, the maximum reach of the system was vastly increased over the EDC case by 85% to 5890 km, which represents the largest ever reported gain in reach due to digital back-propagation. The optimum per channel launch power for the 7-channel signal also increased to −3.5 dBm, which was slightly lower than the predicted optimum launch power obtained through numerical simulations (solid line). As illustrated in Fig. 5(a), excellent agreement was achieved between the experimental and simulation results in the linear region of the reach curve, however the simulation outperformed the experiment in the nonlinear region. The reduced level of performance that was experimentally demonstrated is attributed to the non-ideal compensation of the combined response of both the transmitter and receiver subsystems, which has a greater impact on performance as the DBP bandwidth increases.

The performance of all seven WDM channels, when only EDC was performed and for full bandwidth MC-DBP, at a maximum transmission reach of 5890 km and a per-channel launch power of −3.5 dBm, is illustrated in Fig. 5(b). The characteristic degradation in BER performance, caused by the receiver ENOB, was again experienced for the outer WDM channels. For the EDC only scenario, the received BER varied from 1.3·10^−1^ (CH: −3) to 8.5·10^−2^ (CH: 0), therefore a SD-FEC code with 100% OH was required to correct the BER of the worst performing channel to below 4.7·10^−3^. When combined with the OH required for the outer HD-FEC code, this provided an ISD of 3.77 b/s/Hz. However, when MC-DBP was employed, the received BER was significantly reduced, with the worst performing channel achieving a BER of 3.66·10^−2^, which enabled the total concatenated FEC OH to be reduced to 27.5%. This provided a super-channel ISD of 6.28 b/s/Hz and a SEDP of 36989(b·km)/s/Hz. Therefore, at this distance and launch power, the use of MC-DBP increased the ISD by 2.51 b/s/Hz.

From Fig. 5(b), it is evident that a 20% SD-FEC overhead is not required for all WDM channels when using MC-DBP. Therefore, the ISD could be improved if multiple coding rates were used. For example, a low OH code could be employed for the central three WDM channels (−1, 0, +1), while a higher code rate could be employed for the outer channels (−3, −2, +2, +3). Alternatively, low-density parity check (LDPC) codes with rates adapted to each individual channel could be employed, thereby achieving the maximum ISD. The improvement in ISD as the MC-DBP bandwidth is increased, at a transmission distance of 5890 km and for a per channel launch power of −3.5 dBm, is displayed in Table 1. The performance was analysed when only one SD-FEC code rate was used (to match the worst performing WDM channel) and when the coding rate was optimised for each WDM channel.

For a single SD-FEC code rate, the ISD of the DP-16QAM super-channel increased by approximately 2 b/s/Hz, relative to the EDC only case, when SC-DBP (10 GHz BW) was employed in the coherent receiver. The ISD was further increased from 5.80 b/s/Hz to 6.28 b/s/Hz when full MC-DBP (70 GHz BW) was carried out. However, as the BER deteriorated towards the edge comb channels, the gain in ISD as a function of MC-DBP bandwidth was limited by the worst performing channel. Therefore, the maximum possible ISD for the super-channel was not reached. When the SD-FEC code rate was optimised for each WDM channel, the maximum ISD increased to 6.60 b/s/Hz, thus providing a SEDP of 38874(b·km)/s/Hz.

## Discussion

**Figure 6:**
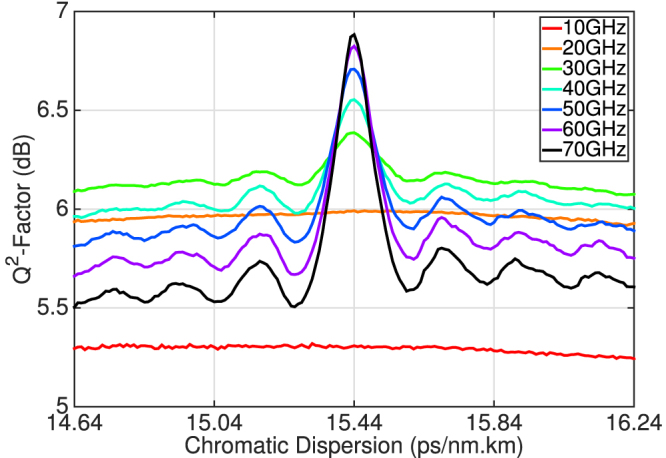
Sensitivity of MC-DBP gain to fibre chromatic dispersion. Received Q^2^-factor as a function of the MC-DBP bandwidth and the chromatic dispersion compensation used in the offline DSP for the central WDM channel at a transmission distance of 5890 km and at a launch power of −3.5 dBm.

Although MC-DBP has provided a record increase in transmission reach of 85%, there are significant challenges associated with this technique, in terms of complexity and the sensitivity of DBP performance to linear channel impairments. Fig. 6 illustrates the received Q^2^-factor as a function of the fibre chromatic dispersion (CD) parameter used in the MC-DBP offline signal processing, for the central WDM channel at a transmission distance of 5890 km and at launch power of −3.5 dBm. The received Q^2^-factor varies by approximately 0.05 dB for DBP bandwidths of 10 GHz and 20 GHz respectively, however as the bandwidth increases the sensitivity to dispersion also increases. The variation in Q^2^-factor for a 30 GHz DBP bandwidth was 0.3 dB, which increased significantly by 1 dB as the MC-DBP bandwidth approached 70 GHz. For the maximum DBP bandwidth, the tolerance to CD to incur a Q^2^-factor penalty less than 0.5 dB was ±0.05 ps/(nm·km). Such a light sensitivity to the variation in CD may limit the performance of MC-DBP in a practical system that is comprised of a series of concatenated fibre links with non-identical CD parameters.

**Figure 7:**
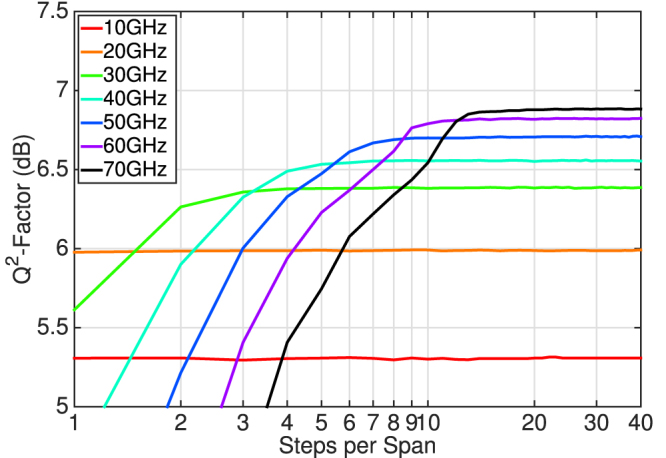
MC-DBP gain as a function of the number of nonlinear steps per span. Received Q^2^-factor as a function of the MC-DBP bandwidth and the number of DBP steps per fibre span for the central WDM channel at a transmission distance of 5890 km and at a launch power of −3.5 dBm.

When considering MC-DBP for fibre nonlinearity compensation, it is important to investigate the number of required nonlinear steps per fibre span, in order to evaluate the associated complexity as a function of DBP bandwidth^20^,^21^. Fig. 7 illustrates the received Q^2^-factor of the central WDM channel at the maximum transmission distance of 5890 km, as a function of both the MC-DBP bandwidth and the number of equally spaced nonlinear steps per span. It is evident that one step per span is sufficient for a MC-DBP bandwidth of 10 GHz or 20 GHz, as the received Q^2^-factor remained constant as the number of steps was increased. Two steps per span were required to achieve a Q^2^-factor gain as the DBP bandwidth was increased to 30 GHz, with a steady state value of 6.4 dB achieved after approximately four steps per span. As the DBP bandwidth increased, there was a corresponding increase in the required number of nonlinear steps per span in order to negate a Q^2^-factor penalty. For the full 7-channel MC-DBP bandwidth of 70 GHz, approximately 20 steps per span were required in order to achieve the maximum gain in the received Q^2^-factor. For an optical network employing lumped amplification, it may be possible to reduce the required number of steps per span for each DBP bandwidth if logarithmically spaced steps were considered^22^.

It can be concluded that a MC-DBP bandwidth of 30 GHz, which incorporates the channel of interest and its two nearest neighbours, provides a good trade-off between performance and complexity. The lower back-propagation bandwidth also demonstrated a lower sensitivity to the estimated chromatic dispersion of the fibre link, achieved a mean ISD of 6.37 b/s/Hz across the super-channel and only required ~4 nonlinear steps per span to achieve an optimum level of performance. However, it is evident that there is a diminishing return in performance gain as a function of increased MC-DBP bandwidths above 30 GHz. This is currently an experimental limitation that is attributed to the non-ideal compensation of the combined response of the transmitter and receiver sub-systems and results in reduced transmission reach relative to that achieved through numerical simulations, as shown in Fig. 5. Therefore, it may be possible to achieve an enhanced level of performance for MC-DBP bandwidths greater than 30 GHz, in terms of transmission reach and ISD, if the performance gap between numerical simulations and experimental measurements can be breached.

## Methods

### Analytical GN Model

Analytical simulations were based on the perturbative Gaussian noise (GN) model^16^, which assumes that the effect of nonlinear interference (NLI) on Nyquist spaced WDM signals in a dispersion uncompensated transmission system can be modelled as additive Gaussian noise. The BER of the system depends on the OSNR, which is modified to include the NLI noise and is defined as: 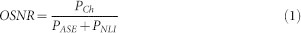
where P_Ch_ is the optical launch power per channel, P_ASE_ is the linear ASE noise of the EDFA in each fibre span and P_NLI_ is the nonlinear interference based on the GN model. The NLI noise is expressed in a closed form approximation: 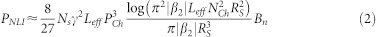
where N_s_ is the number of spans, γ is the nonlinear coefficient of the fibre, β_2_ is the fibre dispersion parameter, L_eff_ is the fibre effective length, N_Ch_ is the number of WDM channels, R_s_ is the symbol rate of the transmission signal and B_n_ is the reference bandwidth of the noise (0.1 nm).

### Numerical Simulation Model

Numerical simulations were based on the split-step solution of the NLSE and were implemented with a temporal resolution of 16 samples per symbol. Practical experimental constraints were also included; for example the DAC used in the digital transmitter had a resolution of 6-bits, but the ENOB was set to 4-bits at a frequency of 10 GHz, in order to match that of the Micram DACs used in the experiment. The single mode fibre was simulated based on the split-step Fourier method and the fibre characteristics were as follows: fibre length of 81.8 km, span loss of 13.4 dB, chromatic dispersion of 16.3 ps/(nm·km), dispersion slope of 0.06 ps/nm^2^/km, PMD coefficient of 0.1 ps/√km, nonlinear coefficient of 0.85 W^−1^/km and 160 steps per span. The EDFA noise figure was 4.5 dB. In the receiver, a 5th order Bessel filter with a 3 dB bandwidth of 63 GHz was applied to the signals to emulate the analogue bandwidth of the digital oscilloscope used in the experiment. Finally, the BER was calculated using 2^17^ symbols, with a PRBS of length 2^15^−1. The receiver DSP was the same as that used in the experiment.

### Digital Signal Processing (DSP)

#### 1. Linear Impairment Compensation

The received signals (sampled at 160GS/s) were initially corrected for receiver skew imbalance and normalised to overcome the varying responsitivities of the 70 GHz balanced photodiodes within the coherent receiver. Chromatic dispersion compensation was subsequently performed before the signals were resampled to 2 samples per symbol and filtered using a matched RRC filter. The signals were equalised using a 21-tap (T/2-spaced) radius directed equaliser^23^, with the constant modulus algorithm^24^ equaliser used for pre-convergence. The intermediate frequency was estimated and removed using a 4^th^-order nonlinearity algorithm^25^. The carrier phase was estimated per polarisation using a decision directed phase estimation algorithm and the complex field was averaged over a 64 T-spaced sliding window to improve the estimate^26^. Gram-Schmidt orthogonalisation^27^ was performed in order to correct for sub-optimal phase bias in the transmitter IQ modulators (which occurred over time due to temperature variations) and the maximum likelihood k-means clustering algorithm^28^ was employed to perform hard decisions. Finally the bit error rate was counted using 2^17^ symbols and the Q^2^-factor was subsequently calculated from the recorded BER.

#### 2. Multi-Channel Digital Back-Propagation

When employing digital back-propagation, the received signals were corrected for receiver skew imbalance and normalised. An ideal brick wall filter was employed to select the desired signal bandwidth for multi-channel digital back-propagation. For single channel DBP the bandwidth was set to 10 GHz, which increased up to 70 GHz for full MC-DBP. DBP was carried out using the split-step Fourier method to form a numerical solution to the Manakov equation. This model accounts for the residual birefringence of the fibre and the effect that this has on the state of polarisation and nonlinearity within the fibre. The number of steps per span was 40 (which was varied for analysis in Fig. 7), with a symmetric split step for chromatic dispersion compensation. After DBP, the complex signals were resampled to 2S/sym, before being filtered using a matched RRC filter. The remaining DSP blocks were identical to that used for the linear equalisation.

### SD-FEC

**Figure 8:**
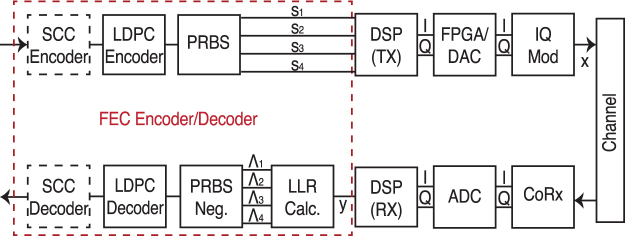
Block diagram of concatenated FEC for one polarisation state.

The proposed FEC scheme is a concatenation of an outer hard decision staircase code (SCC)^29^ and an irregular repeat-accumulate (IRA) inner LDPC code, as shown in Fig. 8. The inner IRA LDPC codes are those proposed by the second generation satellite digital video broadcasting standard^30^ with rates R_c_∈{1/3, 1/2, 3/4, 9/10}, which lead to FEC overheads of 200%, 100%, 33% and 11%. To obtain additional rates to those proposed in the standard, the codes were punctured via pseudorandom puncturing patterns. This provided a larger family of code rates, which enabled the FEC overhead to be tailored to each of the received WDM channels. The inner LDPC code was implemented offline in Matlab. An outer SCC code with a rate R = 16/17 (6.25% OH) was assumed, as this code produces a post-FEC BER of 10^−15^ for a pre-FEC BER of 4.7·10^−3^ 19.

At the transmitter (top half of Fig. 8), four decorrelated pseudo-random binary sequences (s_1_-s_4_) of length 2^15^−1 were generated and combined to provide two 4-level driving signals. This provided the in-phase (I) and quadrature (Q) components that were applied to the IQ modulator to generate the transmitted symbols (x). The received symbols (y) were obtained from the output of the receiver side DSP and were used to compute logarithmic likelihood ratios (LLRs). Four LLRs were calculated for each received complex symbol per polarisation via: 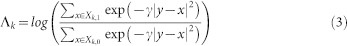
where γ is the SNR and X_k,b_ is the set of constellation symbols labelled with a bit b∈{0, 1}, at bit position k ∈ {1, 2, 3, 4}.

At the receiver (bottom half of Fig. 8), it was assumed that the all-zero codeword of length 64800 (for the un-punctured code) was transmitted, therefore PRBS negation was employed. This inverted the sign of the LLR each time a 1 bit was transmitted. LDPC decoding was subsequently performed based on the un-quantised LLRs and a maximum of 50 iterations of the message-passing algorithm. If the hard-decisions from the LDPC decoder were below the threshold for the SCC (4.7·10^−3^), a post-FEC BER of 10^−15^ was assumed to have been achieved.

## Author Contributions

R.M. and K.S. constructed the experimental setup. R.M., L.G. and M.S. recorded the experimental measurements. T.X. carried out the numerical simulations and performed the analytical calculations. A.A. designed and simulated the forward error correction scheme. R.M., S.J.S., B.C.T., R.I.K. and P.B. contributed to developing the research ideas and were involved in the discussion of results. RM wrote the main manuscript text and prepared the figures. All authors reviewed the manuscript.

## Figures and Tables

**Table 1:** Required FEC overhead and resulting ISD as a function of the MC-DBP bandwidth for a transmission distance of 5890 km, when a single SD-FEC code rate is employed and alternatively when the code rate was optimised for each WDM channel. The FEC OH and mean FEC OH are a combination of both the SD-FEC OH and the 6.25% OH required for the outer HD-FEC code

	Single SD-FEC Code Rate		Optimised SD-FEC Code Rate	
MC-DBP BW	FEC OH	ISD (b/s/Hz)	Mean FEC OH	Mean ISD (b/s/Hz)
EDC Only	112.5%	3.77	94.81%	4.11
10 GHz	38.02%	5.80	33.66%	5.99
20 GHz	32.81%	6.02	26.76%	6.31
30 GHz	31.22%	6.10	25.63%	6.37
40 GHz	31.22%	6.10	24.14%	6.44
50 GHz	29.63%	6.17	22.97%	6.51
60 GHz	27.5%	6.28	22.22%	6.55
70 GHz	27.5%	6.28	21.23%	6.60
